# Case Report: Secukinumab Experience in a Patient With Coexistence of Systemic Lupus Erythematosus, Ankylosing Spondylitis, and Antiphospholipid Antibody Syndrome

**DOI:** 10.1155/crrh/4852681

**Published:** 2026-05-28

**Authors:** Enes Basaran, Busra Yaprak Bayrak, Duygu Temiz Karadag, Ayten Yazici, Ayse Cefle

**Affiliations:** ^1^ Department of Internal Medicine, Division of Rheumatology, Faculty of Medicine, Kocaeli University, Kocaeli, Turkey, kocaeli.edu.tr; ^2^ Department of Pathology, Faculty of Medicine, Kocaeli University, Kocaeli, Turkey, kocaeli.edu.tr

**Keywords:** ankylosing spondylitis, antiphospholipid syndrome, IL-17 inhibitor, secukinumab, systemic lupus erythematosus

## Abstract

Systemic lupus erythematosus (SLE) is a multisystem autoimmune disease characterized by autoantibody production and immune complex deposition. Antiphospholipid syndrome (APS) is frequently associated with SLE and is characterized by arterial or venous thrombosis and pregnancy morbidity in the presence of persistent antiphospholipid antibodies. The coexistence of ankylosing spondylitis (AS) with SLE and APS is extremely rare. A 38‐year‐old woman was admitted to our clinic with gangrene of the right fifth toe and swelling of both knees. She had a previous diagnosis of AS based on inflammatory low back pain and radiographic evidence of bilateral Grade 4 sacroiliitis. Laboratory investigations revealed positive antinuclear antibody, anti‐double‐stranded DNA, and antiphospholipid antibodies, including lupus anticoagulant and anti‐cardiolipin IgG. Renal biopsy performed due to proteinuria demonstrated mesangioproliferative lupus nephritis. Based on clinical, laboratory, and histopathological findings, the patient was diagnosed with coexisting AS, SLE, and APS. Considering the coexistence of these autoimmune diseases and the patient’s previous exposure to anti‐tumor necrosis factor therapy, treatment with the interleukin‐17 inhibitor secukinumab was initiated. The patient showed significant improvement in inflammatory back pain and arthritis during follow‐up. To our knowledge, this is one of the rare reported cases of coexistence of AS, SLE, and APS treated with secukinumab.

## 1. Introduction

Systemic lupus erythematosus (SLE) is a multisystem autoimmune disease characterized by autoantibody production and immune complex deposition, leading to organ damage [1]. Antiphospholipid syndrome (APS), which may occur in up to 20%–40% of patients with SLE, increases the risk of arterial and venous thrombosis as well as pregnancy morbidity through prothrombotic mechanisms [2]. Ankylosing spondylitis (AS) is a chronic inflammatory disease primarily affecting the axial skeleton and is strongly associated with HLA‐B27 and IL‐17–mediated pathways [3]. While SLE and APS commonly coexist, their simultaneous presence with AS is exceptionally rare, reflecting potentially distinct immunopathogenic mechanisms and posing diagnostic and therapeutic challenges.

In this report, we present a patient with coexisting SLE, APS, and AS, a rare overlap described in only a limited number of cases in the literature. Additionally, secukinumab, an IL‐17A inhibitor approved for AS, was used as part of the management strategy. This case highlights the clinical complexity of overlapping autoimmune diseases with different immunopathogenic mechanisms and suggests a potential role for IL‐17 inhibition in such rare scenarios.

## 2. Case Presentation

A 38‐year‐old woman was referred to our clinic because of gangrene of the right fifth toe and swelling of both knees. The patient had a previous diagnosis of AS, which had been established several years earlier based on inflammatory low back pain and radiographic evidence of sacroiliitis. She had initially been treated with etanercept but showed inadequate response and was subsequently switched to golimumab, which she used for approximately 10 years. During the last year, she had been followed only with nonsteroidal anti‐inflammatory drugs (NSAIDs) after discontinuing golimumab on her own.

The patient also had a history of one premature birth at 28 weeks of gestation. Apart from inflammatory low back pain and peripheral joint symptoms, the systemic review was otherwise unremarkable.

**FIGURE 1:**
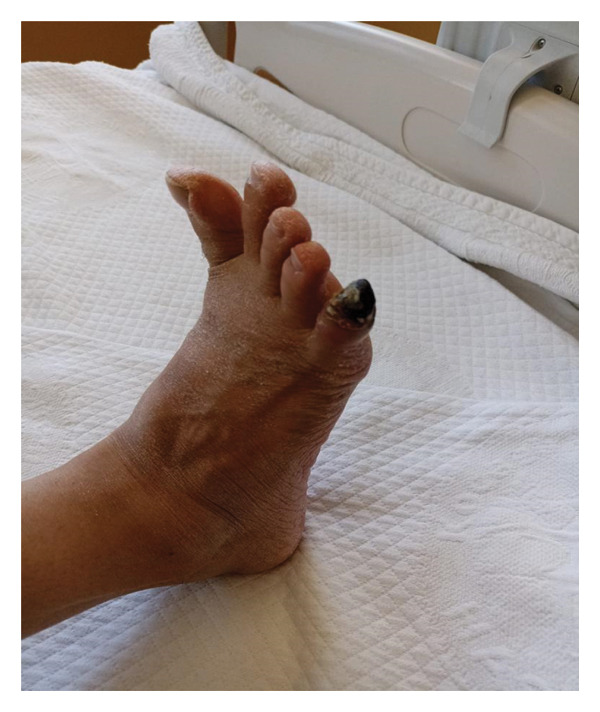
Focal necrotic lesion involving the distal phalanx of the 5th toe, right foot, with well‐demarcated borders.

Physical examination revealed bilateral knee swelling and tenderness. The hand‐to‐floor distance was 25 cm, and the modified Schober’s test showed limited lumbar flexion (3 cm). Gangrene of the right fifth toe was observed (Figure 1). Examination of other systems did not reveal any pathological findings.

**FIGURE 2:**
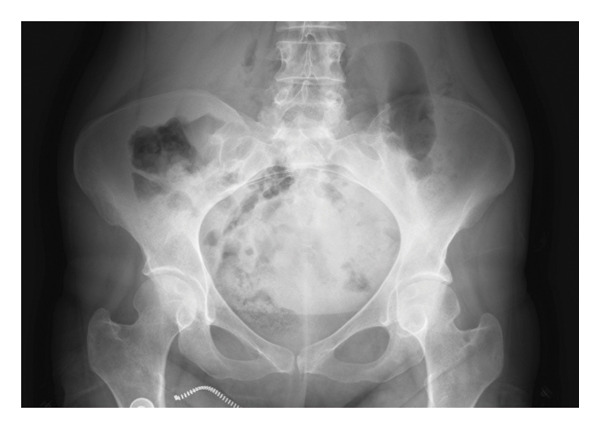
Bilateral Grade 4 sacroiliitis.

Radiographic evaluation of the sacroiliac joints demonstrated bilateral Grade 4 sacroiliitis (Figure 2). Based on inflammatory low back pain and limitation of lumbar flexion together with radiographic findings, the patient fulfilled the modified New York criteria and the Assessment of Spondyloarthritis International Society (ASAS) classification criteria for AS.

Lower extremity arterial Doppler ultrasonography did not reveal any major arterial obstruction. Laboratory analysis showed antinuclear antibody (ANA) positivity with a speckled pattern at a titer of 1:320. Anti‐double‐stranded DNA (anti‐dsDNA) and antihistone antibodies were positive in the extractable nuclear antigen (ENA) panel. Anti‐dsDNA level was 318 IU/mL. In addition, antiphospholipid antibody testing revealed anti‐cardiolipin IgG > 120 U/mL, lupus anticoagulant positivity, and anti‐β2 glycoprotein‐I IgG of 73.3 U/mL. Direct Coombs test was also positive. A summary of the patient’s other laboratory parameters is presented in Table 1. Based on the clinical and immunological findings, the patient fulfilled the American College of Rheumatology (ACR) and Systemic Lupus International Collaborating Clinics (SLICC) classification criteria for SLE.

**TABLE 1:** Laboratory findings and disease activities of the patient at the time of diagnosis, 3rd month, and 12th month of treatment.

Laboratory parameter	Result (at the time of diagnosis)	Result (3rd month of treatment)	Result (12th month of treatment)	Reference range
Glucose	78 mg/dL	65 mg/dL	71 mg/dL	74–106 mg/dL
Urea	33.8 mg/dL	15.1 mg/dL	19.6 mg/dL	16–48 mg/dL
Creatinine	0.4 mg/dL	0.46 mg/dL	0.4 mg/dL	0.7–1.2 mg/dL
AST	17.9 U/L	17.3 U/L	18.2 U/L	1–40 U/L
ALT	13.3 U/L	6.9 U/L	13.3 U/L	1–40 U/L
LDH	176 U/L	171 U/L	141 U/L	135–225 U/L
WBC	8.16 × 10^3^/μL	7.56 × 10^3^/μL	8.97 × 10^3^/μL	3.4–10 × 10^3^/μL
Neu	6.02 × 10^3^/μL	4.6 × 10^3^/μL	5.5 × 10^3^/μL	1.4–7.3 × 10^3^/μL
Lym	1.3 × 10^3^/μL	1.9 × 10^3^/μL	2.3 × 10^3^/μL	1–3.1 × 10^3^/μL
Hgb	10.6 g/dL	13.6 g/dL	11 g/dL	12–16.6 g/dL
Plt	296 × 10^3^/μL	343 × 10^3^/μL	327 × 10^3^/μL	172–380 10^3^/μL
C3	1 g/L	1.02 g/L	1.08 g/L	0.9–1.8 g/L
C4	0.11 g/L	0.13 g/L	0.11 g/L	0.1–0.4 g/L
CRP	13.6 mg/L	3.49 mg/L	2 mg/L	< 5 mg/L
ESR	21 mm/h	11 mm/h	9 mm/h	< 20 mm/h
ANA	Speckled 1/320			
ENA	Ds dna 1+, histones 1+			
Anti‐dsDNA (ELISA)	318.33	99.59	53.17	< 100
Direct Coombs	Positive	Positive	Negative	
Lupus anticoagulant	Positive	Positive		
AC‐Ig M	Negative	Negative		
AC‐Ig G	> 120 U/mL	> 120 U/mL		
aβ2 GPI Ig M	Negative	Negative		
aβ2 GPI Ig G	73.3 U/mL	63.2 U/mL		
24‐h urinary protein excretion	416.8 (mg/day)	61.8 (mg/day)	78.2 (mg/day)	0–140 mg/day
SLEDAI‐2K	18	8	0	
BASDAI	6.2	2.8	1.5	

*Note:* AST, aspartate aminotransferase; ALT, alanine aminotransferase; LDH, lactate dehydrogenase; Neu, neutrophil; Lym, lymphocyte; Hgb, hemoglobin; Plt, platelet; anti‐dsDNA, double‐stranded antibodies; C3, complement component 3; C4, complement component 4; ENA, extractable nuclear antigen antibodies; aC, anticardiolipin antibodies; aβ2 GPI, anti‐β2‐glycoprotein‐I antibodies.

Abbreviations: ANA, antinuclear antibody; BASDAI, Bath Ankylosing Spondylitis Disease Activity Index; CRP, C‐reactive protein; ESR, erythrocyte sedimentation rate; SLEDAI‐2K, the Systemic Lupus Erythematosus Disease Activity Index 2000; WBC, white blood count.

**FIGURE 3:**
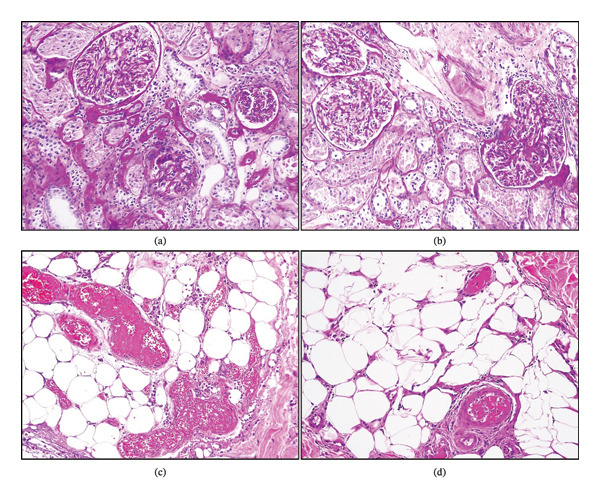
(a, b) Significant mesangial cell increase and expansion and focal basement membrane thickening in glomeruli, degenerative changes in tubules (PAS, × 200). (c, d) Diffuse fibrin thrombus, focal septal necrosis, and inflammation in small‐ and medium‐sized vessels in the deep dermis and subcutaneous fatty tissue (H&E, × 200).

A biopsy was obtained from the necrotic lesion of the right fifth toe. Histopathological examination revealed diffuse fibrin thrombi in small‐ and medium‐sized vessels within the deep dermis, focal septal necrosis, and inflammatory infiltration in the subcutaneous adipose tissue (Figure 3a). These findings were compatible with APS.

Because 24‐hour urine analysis demonstrated proteinuria of 416 mg and microscopic hematuria, a renal biopsy was performed. Histopathological examination revealed mesangial cell proliferation and focal basement membrane thickening. Immunofluorescence microscopy demonstrated deposition of IgG and IgM. These findings were consistent with mesangioproliferative lupus nephritis (Class II) (Figure 3b).

A thrombosis panel was performed to investigate possible causes of arterial thrombosis, and no additional abnormalities were detected. Transthoracic echocardiography did not reveal any valvular vegetations.

The history of premature birth at 28 weeks was considered likely related to APS in the context of persistent antiphospholipid antibody positivity.

Based on the clinical, laboratory, and histopathological findings, the patient was diagnosed with coexisting AS, SLE, and APS.

Discontinuation of TNF inhibitor therapy alone was not sufficient to control the patient’s symptoms, and specific treatment for SLE, including hydroxychloroquine and glucocorticoids, was required.

For the management of SLE nephritis (Class II) and APS, hydroxychloroquine, low‐dose glucocorticoids, and anticoagulation therapy were initiated. Because the patient continued to experience inflammatory low back pain despite NSAID therapy and had active peripheral arthritis, biological therapy for AS was considered. Taking into account the coexistence of SLE and APS and the patient’s previous exposure to anti‐TNF agents, secukinumab, an interleukin‐17 inhibitor, was initiated.

Disease activity scores including BASDAI and SLEDAI were recorded at baseline and during follow‐up at the 3rd and 12th months of treatment (Table 1). The patient continues to be followed in our clinic under ongoing treatment.

Written informed consent was obtained from the patient for publication of this case report.

## 3. Discussion

SLE is a chronic multisystem autoimmune disease characterized by autoantibody production and immune complex deposition that can affect various organs and systems [1]. APS is frequently associated with SLE and is characterized by arterial or venous thrombosis and/or pregnancy morbidity in the presence of persistent antiphospholipid antibodies [2]. The coexistence of AS with SLE, however, is considered extremely rare.

AS is a chronic inflammatory disease primarily affecting the axial skeleton and is strongly associated with HLA‐B27 and the IL‐17 inflammatory pathway [3]. Although sacroiliitis may occasionally be observed in patients with SLE, the coexistence of definite AS and SLE has been reported only in a limited number of cases in the literature [4, 5]. The first description of this overlap was reported by Nashel et al. in 1982 [4], and only a small number of additional cases have been published since then [5–7].

One of the most important aspects of our case is the chronological sequence of the diagnoses. In many previously reported cases, sacroiliitis developed during the course of SLE, raising the possibility that inflammatory back pain may represent a musculoskeletal manifestation of lupus rather than true spondyloarthritis [5, 8]. In contrast to previously reported cases where sacroiliitis developed during the course of SLE, our patient had a well‐established diagnosis of AS long before the onset of SLE and APS. This chronological sequence supports the coexistence of two distinct autoimmune diseases rather than a musculoskeletal manifestation secondary to SLE.

An important diagnostic consideration in our patient was the possibility of TNF inhibitor–induced drug‐induced lupus (DIL). Although anti‐TNF–associated DIL may present with anti‐dsDNA positivity, it is typically characterized by milder clinical features and rarely involves major organ manifestations such as lupus nephritis. In our case, the presence of biopsy‐proven lupus nephritis, concomitant APS, and persistently elevated anti‐dsDNA levels, together with the lack of clinical resolution after discontinuation of TNF inhibitor therapy, strongly supported a diagnosis of de novo SLE rather than DIL.

The presence of APS in our patient further increases the complexity of this clinical overlap. APS occurs in approximately 20%–40% of patients with SLE and significantly increases the risk of arterial and venous thrombosis [2]. Digital ischemia and gangrene represent rare but severe manifestations of APS‐related vascular involvement [9, 10]. In our patient, histopathological examination of the necrotic lesion revealed fibrin thrombi in small and medium‐sized vessels, consistent with thrombotic microangiopathy typically observed in APS.

Another important finding in our case was the presence of lupus nephritis. Renal biopsy demonstrated mesangioproliferative lupus nephritis (Class II), which represents a relatively mild form of renal involvement in SLE. The coexistence of lupus nephritis with APS‐related vascular manifestations has been described previously and may reflect the complex interaction between immune‐mediated inflammation and thrombotic mechanisms in SLE [1, 2].

The coexistence of multiple autoimmune diseases in a single patient has been increasingly recognized, with rare reports describing complex overlap syndromes involving conditions such as SLE, APS, AS, rheumatoid arthritis, and Sjögren’s syndrome. These cases highlight the challenges in diagnosis and management of patients with overlapping autoimmune disorders [11].

The optimal treatment strategy in patients with overlapping autoimmune diseases remains challenging. Tumor necrosis factor inhibitors are widely used in the treatment of AS; however, these agents have been associated with the development of lupus‐like syndromes in some patients [12]. Although our patient had previously received anti‐TNF therapy for AS, SLE was diagnosed many years later, making DIL less likely.

Considering the coexistence of AS, SLE, and APS, we preferred treatment with secukinumab, an interleukin‐17 inhibitor, for the management of axial disease. The IL‐17 pathway plays an important role in the pathogenesis of spondyloarthritis [13]. Interestingly, IL‐17 has also been implicated in the immunopathogenesis of SLE, suggesting that these diseases may share partially overlapping inflammatory pathways [14]. However, the role of IL‐17 inhibition in SLE remains unclear, and isolated cases of lupus development during secukinumab therapy have also been reported [15].

Our case therefore highlights several important clinical issues. First, although rare, the coexistence of AS and SLE should be considered in patients presenting with inflammatory back pain and lupus‐related manifestations. Second, APS‐related vascular complications such as digital gangrene may be the presenting manifestation leading to the diagnosis of SLE. Finally, the management of patients with overlapping autoimmune diseases requires careful therapeutic selection, particularly when biological agents are considered.

In conclusion, we report a rare case of coexistence of AS, SLE, and APS presenting with digital gangrene and lupus nephritis. This case emphasizes the importance of recognizing possible overlaps between autoimmune diseases and highlights the challenges in therapeutic decision‐making in such complex clinical scenarios.

## Author Contributions

E.B. contributed to the conception and design of the study, patient follow‐up, data collection, literature review, manuscript drafting, and final revision of the manuscript.

B.Y.B. contributed to the histopathological evaluation and interpretation of pathological findings.

D.T.K. contributed to data interpretation, critical revision of the manuscript, and supervision of the clinical process.

A.Y. contributed to the clinical evaluation of the patient, interpretation of rheumatological findings, and critical revision of the manuscript.

A.C. contributed to the study conception, supervision, and final critical review of the manuscript.

## Funding

The authors received no financial support for the research, authorship, and/or publication of this article.

## Disclosure

All authors have read and approved the final version of the manuscript.

## Ethics Statement

Ethical approval was not required for this case report in accordance with local institutional policies. Written informed consent was obtained from the patient for publication of this case report and accompanying images.

## Conflicts of Interest

The authors declare no conflicts of interest.

## Data Availability

All data generated or analyzed during this study are included in this published article. Further patient‐related data are not publicly available due to privacy restrictions but are available from the corresponding author upon reasonable request.
